# Can Sleep Be Used as an Indicator of Overreaching and Overtraining in Athletes?

**DOI:** 10.3389/fphys.2018.00436

**Published:** 2018-04-24

**Authors:** Michele Lastella, Grace E. Vincent, Rob Duffield, Gregory D. Roach, Shona L. Halson, Luke J. Heales, Charli Sargent

**Affiliations:** ^1^Appleton Institute for Behavioural Science, School of Health, Medical and Applied Sciences, Central Queensland University, Adelaide, SA, Australia; ^2^Sport and Exercise Discipline Group, University of Technology Sydney, Sydney, NSW, Australia; ^3^Department of Physiology, Australian Institute of Sport, Canberra, ACT, Australia; ^4^School of Health, Medical and Applied Sciences, Central Queensland University, Rockhampton, QLD, Australia

**Keywords:** intensified training, recovery, exercise, athlete monitoring, training load, training scheduling

## Introduction

To achieve optimal athletic performance and competition readiness, it is crucial to balance the highest appropriate training stimulus with sufficient recovery. Excessive and/or progressive increases in training load are integral to improving athletic performance (Halson, 2014). However, increased training loads and/or inadequate recovery can result in maladaptation to training, and if continued, can lead to the development of overreaching/overtraining (Meeusen et al., 2013; Cadegiani and Kater, 2017). In terms of recovery, sleep is an essential component of an athlete's recuperation due to its physiological and psychological restorative effects (Dinges et al., 1997; Pejovic et al., 2013). Sleep quantity and quality declines following augmented increases (+30%) in training load (Hausswirth et al., 2014), and poor sleep is a common complaint among overreached and/or overtrained athletes (Wall et al., 2003). Regardless of whether reduced sleep is a cause or effect of overreaching and/or overtraining, it is possible that measures of sleep could serve as an indicator of the presence of overreaching and/or overtraining. This opinion article will examine the current research underpinning the relationship between insufficient sleep and the development of overreaching/overtraining, describe the implications for practitioners (e.g., sport and exercise scientists, coaches), and identify areas for future research.

## Training load, overreaching/overtraining, and sleep

Overreaching occurs when there is an imbalance between training and recovery (Halson and Jeukendrup, 2004). Overreaching can be actively prescribed into a periodised training program to induce improvements in athletic performance (termed “functional overreaching”) (Halson and Jeukendrup, 2004; Kellmann, 2010). However, further accumulation of training volume or intensity—when combined with insufficient or impaired recovery—can lead to “non-functional overreaching” and the eventual development of the overtraining syndrome, resulting in detriments in athletic performance (Meeusen et al., 2013; Cadegiani and Kater, 2017).

Sleep is considered a vital component for both physical and mental recovery from exercise (Robson-Ansley et al., 2009; Chase et al., 2017) and is believed to be the single best recovery strategy available to athletes (Halson, 2008). Interestingly, sleep disturbances are frequently reported as one of the many symptoms of overreaching/overtraining (Kellmann, 2010; Meeusen et al., 2013). For example, sleep efficiency (a measure of sleep quality) is significantly different between non-overreached and overreached swimmers (95 vs. 82%) (Wall et al., 2003). These results suggest that disturbances in sleep occur in parallel with overreaching/overtraining, but it is also possible the relationship is coincidental (Hausswirth et al., 2014; Fullagar et al., 2015).

There are a number of potential factors that may contribute specifically to inadequate sleep in overreached/overtrained athletes. Two key factors are (i) training load, and (ii) scheduling of training and competition. During periods where training loads are high, some athletes report difficulties falling asleep, restlessness during sleep, and heavy legs during sleep (Fry et al., 1994; Halson et al., 2006). Increases in training load have been negatively associated with sleep quantity/quality in female youth soccer players (Watson et al., 2016) and Australian rules football players (Pitchford et al., 2017). While the physiological mechanism underlying sleep disturbance during periods of overreaching/overtraining are unclear, changes in immune function may provide a potential explanation. Indeed, overreached athletes showed objective signs of moderate sleep disturbance and higher prevalence of upper respiratory tract infections when compared to non-overreached athletes (Hausswirth et al., 2014). Following increases in training load, several aspects of innate and adaptive immunity are depressed (e.g., marked reductions in neutrophil function, lymphocyte proliferation and number of circulating T cells) (Halson et al., 2003; Cadegiani and Kater, 2017). Depressed immune response, often leading to infection, has been shown to impair sleep (Imeri and Opp, 2009). However, further studies are needed to determine whether alterations in immune function as a result of overreaching/overtraining directly lead to sleep disturbance, given that lack of sleep itself also has a direct impact on immune function (Besedovsky et al., 2012). In addition, when an overreached/overtrained athlete is underperforming, this can lead to an increase in stress and anxiety, and negative mood, all of which can adversely impact sleep (Thomsen et al., 2003; Åkerstedt et al., 2007; Petersen et al., 2013). Likewise, reduced sleep also can impair mood and increase stress/anxiety (Dinges et al., 1997; Meerlo et al., 2008). These reciprocal relationships make it difficult to determine the direction of the relationships between sleep, immune function, stress, anxiety, and mood, and how these in turn may lead or contribute to the development of overreaching/overtraining.

Sleep has been shown to vary across the competitive season. In a seminal study employing the gold standard in sleep monitoring, polysomnography, swimmers obtained more slow-wave sleep during the start of season (29%) and peak (29%) training phases, compared to the taper phase (18%) (Taylor et al., 1997). This finding is consistent with the restorative theory of sleep where during slow-wave sleep the body releases growth hormones to stimulate the protein synthesis necessary for body restoration (Adam and Oswald, 1983; Dattilo et al., 2011). In contrast, the number of movements during sleep was significantly greater at the start of the season (46 ± 26) and peak (49 ± 30) training phases, compared to taper (31 ± 19), which is indicative of poor sleep quality (Taylor et al., 1997). Another study utilizing polysomnography reported that overreached triathletes exhibit significant decreases in sleep duration (−5.4%), sleep efficiency (−2.1%) and immobile time (−5.7%) when compared to baseline and taper (Hausswirth et al., 2014). In that study, time in bed and sleep latency remained unchanged, suggesting a decrease in sleep duration and an increase in wake duration during sleep episodes (Hausswirth et al., 2014). Taken together, such results suggest that reduced sleep could cause overreaching/overtraining or could be the result of overreaching/overtraining (Lewis et al., 2015).

The direct impact of an increased training load is often thought of as the primary factor influencing sleep of overreached/overtrained athletes. However, increases in training load are often accompanied by changes in training and competition scheduling, which may influence the amount of time an athlete can spend in bed. The impact of training and competition schedules on athletes' sleep is well established (Roach et al., 2013; Sargent et al., 2014; Lastella et al., 2015a,b). For example, early morning training and competition times have been shown to reduce athletes' sleep duration and increase pre-training fatigue levels (Sargent et al., 2014; Lastella et al., 2015a). Further, in a study of Australian rules football players, evening games were associated with later sleep onset time, shorter time in bed, and less total sleep obtained, when compared to day games (Sargent and Roach, 2016). As such, when designing training programs practitioners need to consider the upcoming competitive schedules, and should also be aware of the implications of training timing on sleep duration and fatigue levels (Sargent et al., 2014). Poorly designed training programs may restrict the opportunity athletes have for sleep, which in turn, may limit recovery between training sessions.

## Implications and areas for future research

Athlete monitoring is crucial to quantify training load, training status, and performance (Saw et al., 2015; Gabbett et al., 2017). Even though many sport and exercise practitioners state that overtraining is the most important reason for athlete monitoring (Taylor et al., 2012), identifying states of overreaching/overtraining is challenging (Jürimäe et al., 2011). Research studies typically evaluate hormones (e.g., insulin like growth factor) to monitor training status, and to determine overreaching and/or overtraining in athletes (Jürimäe et al., 2002; Mäestu et al., 2003, 2006). However, measures of sleep, for example, polysomnography, wrist actigraphy, or sleep diaries may be more feasible, compared to analyzing hormone levels in blood samples for predicting the onset of overreaching/overtraining (Jürimäe et al., 2011). Indeed, mood states questionnaires and reports of subjective sleep complaints (e.g., sleep disturbances and delayed sleep latency) are consistently described as an early marker of maladaptation to training (Urhausen and Kindermann, 2002; Halson and Jeukendrup, 2004). However, it is important to acknowledge that sleep in athletes can be disrupted for a number of reasons including noise, unfamiliar sleep environments, pre-competition anxiety (Lastella et al., 2014a; Juliff et al., 2015), night-time competition (Sargent and Roach, 2016), transmeridian travel (Lastella et al., 2014b; Fowler et al., 2015) and altitude (Roach et al., 2013). Further research is needed to determine the direction of the relationship between sleep disturbances and overreaching/overtraining. In addition, investigating which aspects of training load (volume and/or intensity) contribute to sleep disturbance should be explored.

The ultimate aim is to establish a procedure for practitioners to identify measures that can be used on a regular basis for early detection of overreaching/overtraining. Further studies are needed to investigate the acute relationships between training load and sleep, and to determine whether countermeasures that minimize sleep loss (e.g., appropriate scheduling of training to maximize sleep opportunity, sleep hygiene, nutritional supplements to promote sleep) can minimize the adverse effects on sleep when training loads are high. Using sleep as a tool to identify individuals at risk of developing overreaching/overtraining could reduce the likelihood of performance impairment and injury. However, prospective longitudinal studies are needed, across a range of sports and abilities (e.g., elite, sub-elite), to further elucidate the relationship between sleep and training status across a competitive season. This could be achieved by employing polysomnography at regular intervals across a competitive season where training load gradually increases. Another approach could be to examine the sleep of overtrained athletes and monitor their recovery from overtraining. Such studies would allow for a direct comparison of athletes that do and do not develop overreaching/overtraining across the competitive season. Special considerations should also be given to younger athletes (Winsley and Matos, 2011; Solomon and Weiss Kelly, 2016), as adolescents in particular exhibit a higher physiological need for sleep and experience delays in the timing of sleep onset and awakening compared with adults (Crowley and Carskadon, 2010).

## Conclusion

Non-functional overreaching and overtraining may result from increased training load, leading to impaired athletic performance. Disturbances in sleep are believed to be a key symptom of overreaching/overtraining, which may be a direct result of increased training load, or indirect alterations to training scheduling. Sleep could represent a feasible indicator of determining overreaching/overtraining status, but further research is needed.

## Author contributions

All authors listed have made a substantial, direct and intellectual contribution to the work, and approved it for publication.

### Conflict of interest statement

The authors declare that the research was conducted in the absence of any commercial or financial relationships that could be construed as a potential conflict of interest.
